# Sulfur Metabolism of *Hydrogenovibrio thermophilus* Strain S5 and Its Adaptations to Deep-Sea Hydrothermal Vent Environment

**DOI:** 10.3389/fmicb.2017.02513

**Published:** 2017-12-13

**Authors:** Lijing Jiang, Jie Lyu, Zongze Shao

**Affiliations:** ^1^Key Laboratory of Marine Genetic Resources, Third Institute of Oceanography, State Oceanic Administration, Xiamen, China; ^2^Fujian Key Laboratory of Marine Genetic Resources, Xiamen, China; ^3^Fujian Collaborative Innovation Center of Marine Biological Resources, Xiamen, China; ^4^Laboratory for Marine Biology and Biotechnology, Qingdao National Laboratory for Marine Science and Technology, Qingdao, China

**Keywords:** *Hydrogenovibrio*, *Thiomicrospira*, sulfur oxidation, *DsrEF*, assimilatory sulfate reduction, hydrothermal vent

## Abstract

*Hydrogenovibrio* bacteria are ubiquitous in global deep-sea hydrothermal vents. However, their adaptations enabling survival in these harsh environments are not well understood. In this study, we characterized the physiology and metabolic mechanisms of *Hydrogenovibrio thermophilus* strain S5, which was first isolated from an active hydrothermal vent chimney on the Southwest Indian Ridge. Physiological characterizations showed that it is a microaerobic chemolithomixotroph that can utilize sulfide, thiosulfate, elemental sulfur, tetrathionate, thiocyanate or hydrogen as energy sources and molecular oxygen as the sole electron acceptor. During thiosulfate oxidation, the strain produced extracellular sulfur globules 0.7–6.0 μm in diameter that were mainly composed of elemental sulfur and carbon. Some organic substrates including amino acids, tryptone, yeast extract, casamino acids, casein, acetate, formate, citrate, propionate, tartrate, succinate, glucose and fructose can also serve as carbon sources, but growth is weaker than under CO_2_ conditions, indicating that strain S5 prefers to be chemolithoautotrophic. None of the tested organic carbons could function as energy sources. Growth tests under various conditions confirmed its adaption to a mesophilic mixing zone of hydrothermal vents in which vent fluid was mixed with cold seawater, preferring moderate temperatures (optimal 37°C), alkaline pH (optimal pH 8.0), microaerobic conditions (optimal 4% O_2_), and reduced sulfur compounds (e.g., sulfide, optimal 100 μM). Comparative genomics showed that strain S5 possesses more complex sulfur metabolism systems than other members of genus *Hydrogenovibrio*. The genes encoding the intracellular sulfur oxidation protein (*DsrEF*) and assimilatory sulfate reduction were first reported in the genus *Hydrogenovibrio*. In summary, the versatility in energy and carbon sources, and unique physiological properties of this bacterium have facilitated its adaptation to deep-sea hydrothermal vent environments.

## Introduction

The environments of deep-sea hydrothermal vents are characterized by steep gradients of physical and chemical parameters in the mixing zones between hot vent fluids and cold deep-sea water. Despite these extreme conditions, vent ecosystems develop quite well based on primary production conducted by chemolithoautotrophic microbes that are either free-living or associated with invertebrates as symbionts (Sievert et al., 2008). In these ecosystems, carbon fixation driven by sulfur oxidation is a major process of primary production. In black chimney ecosystems, hydrogen sulfide in hydrothermal fluids generated by seawater-rock interactions in the sub-seafloor (Jannasch and Mottl, 1985) serves as the major energy source for chemolithoautotrophs (McCollom and Shock, 1997). In addition to hydrogen sulfide, elemental sulfur, thiosulfate and polysulfide can be found in both the mixing zones and far away from the vents (Mullaugh et al., 2007; Gartman et al., 2011; Beinart et al., 2015). These partially oxidized inorganic sulfur compounds can be further oxidized by chemolithoautotrophic sulfur-oxidizing bacteria (CSOB).

*Hydrogenovibrio* bacteria are a type of CSOB that were first identified around the Galapagos Rift vents, and originally described as strains of the genus *Thiomicrospira* (Ruby et al., 1981). However, members of the genus *Thiomicrospira* from deep-sea hydrothermal vents were reclassified to the genus *Hydrogenovibrio* based on phylogeny, physiology and morphology during preparation of this manuscript (Boden et al., 2017). To date, several bacteria belonging to this genus have been isolated from deep-sea hydrothermal vent environments and characterized, including strain L-12 (Ruby and Jannasch, 1982), strain TH-55 (Jannasch et al., 1985), strain MA2-6 (Brinkhoff and Muyzer, 1997), strain MA-3 (Wirsen et al., 1998), strain XCL-2 (Ahmad et al., 1999), strain I78 (Takai et al., 2004), strain SP-41(Hansen and Perner, 2014), and strain EPR85 (Houghton et al., 2016). However, only *Thiomicrospira crunogenus* and *Thiomicrospira thermophilus* have been subjected to species description, (Jannasch et al., 1985; Takai et al., 2004), which resulted in their recently being renamed as *Hydrogenovibrio crunogenus* and *Hydrogenovibrio thermophilus* (Boden et al., 2017). In addition, culture-independent methods revealed that bacteria of this genus were predominant members of communities in hydrothermal vent samples, such as the Lost City carbonate chimney and sulfide chimneys in the Southwest Indian Ridge (SWIR) (Brazelton and Baross, 2010; Cao et al., 2014). Thus, members of this genus are proposed to play an important role in sulfur and carbon cycling in hydrothermal vent systems. To date, only one deep-sea hydrothermal vent bacterium in the genus (*H. crunogenus* XCL-2) has had its genome completely sequenced (Scott et al., 2006). Genomic analysis indicated that strain XCL-2 was an obligate chemolithoautotroph, possessing genes encoding a carbon-concentrating mechanism and Calvin-Benson-Bassham (CBB) cycle. Oxidation of reduced sulfur compounds by strain XCL-2 relied on the Sox system and sulfide:quinone reductase (SQR). Moreover, Brazelton and Baross (2010) reported metagenomic sequences derived from a carbonate chimney of the Lost City vent field on the MAR that were highly similar to the genome of strain XCL-2, even though they inhabit different niches in two different hydrothermal vent systems (a black chimney and a white chimney) in two separate oceans. These findings imply that these organisms are highly adaptable in hydrothermal vent systems.

The SWIR is recognized as an ultraslow spreading ridge that has not been thoroughly characterized in terms of geology, geochemistry and ecology. In a previous study, metagenomics analysis revealed that members of the genus *Thiomicrospira*, now reclassified as *Hydrogenovibrio*, were relatively abundant in the black chimney sulfides of the SWIR (Cao et al., 2014). However, defining their roles *in situ* requires more studies of their physiology, metabolism, and adaptation to the deep-sea hydrothermal vents. In this report, we isolated a bacterium of *Hydrogenovibrio* from an active hydrothermal vent chimney on the SWIR that was closely related to *H. thermophilus* (Takai et al., 2004; Houghton et al., 2016). Further analyses were conducted to characterize its physiology, annotate its genome and investigate its sulfur oxidation processes. The results denote its role and adaption to micro-niches in hydrothermal vent environments.

## Materials and methods

### Sample collection, enrichment, and isolation

During the COMRA DY30 oceanic research cruise (March 2014), black chimney samples were collected from an active hydrothermal vent with a remotely operated vehicle (ROV) “*Hai-long II”* from a depth of 2,742 m on the SWIR (49°39′E, 37°47′S; Site 30III-S005-ROV01). Aboard the research vessel *Da-yang Yi-hao*, samples were immediately transferred into 100 ml glass bottles under a gas phase of 100% N_2_ (100 kPa), containing 50 ml sterilized MJ synthetic seawater (Takai et al., 1999) and 0.05% (w/v) sodium sulfide. The suspended slurry was inoculated into MMJHS medium (Takai et al., 2003) under a gas phase mixture of 80% H_2_, 18% CO_2_, and 2% O_2_ (200 kPa), after which the culture was incubated at 28°C. Cells were then purified with the dilution-to-extinction technique using the same medium. The purity of the culture was confirmed by microscopic examination and 16S ribosomal RNA (rRNA) gene sequencing.

### Growth characteristics

The physiological characterization of the isolate was detected on MMJS medium (Takai et al., 2003). After autoclaving, the medium was dispensed into 50 ml serum bottles, then sealed with a butyl-rubber stopper under a gas phase mixture of 80% N_2_, 18% CO_2_ and 2% O_2_ (200 kPa). Unless otherwise stated, the experiments were conducted in triplicate. Bacterial growth was measured by spectrophotometry and direct cell counting using a phase contrast microscope (Eclipse 80i, Nikon, Japan). The optimum growth conditions were tested under various parameters, including different temperatures (20°C, 25°C, 28°C, 30°C, 35°C, 37°C, 40°C, and 45°C), salt concentrations (0, 1, 2, 3, 4% (w/v) NaCl), pH (5.5, 7.0, and 8.0), and oxygen concentrations (gas phase settings of 0, 2, 4, 6, 8, 10, and 20%). In the case of oxygen absence, 10 mM nitrate was added as a potential electron acceptor.

Heterotrophic growth was tested in a NaHCO_3_-minus MMJS medium containing potential organic carbon sources under a gas phase of 4% O_2_: 0.1% (w/v) peptone, yeast extract, tryptone, starch, casein and casamino acids, 5 mM of acetate, formate, citrate, tartrate, succinate, propionate and pyruvate, 5 mM each of 20 amino acids, 0.02% (w/v) sucrose, galactose, glucose, lactose, fructose, maltose and trehalose.

### Oxidation of inorganic sulfur compounds and hydrogen

The ability for sulfur oxidation was tested in the MMJS medium using various sulfur compounds at different concentrations as the sole energy source, including thiosulfate (5, 10, 15, 20, 30 mM), sodium sulfide (50, 100, 200, 400, 800 μM, 1, 2 mM), sulfite (5 mM), elemental sulfur (1% w/v), thiocyanate (5 mM) or tetrathionate (5 mM). Growth characteristics along with sulfur oxidation were examined under the optimal growth conditions with 10 mM Na_2_S_2_O_3_ and 100 μM Na_2_S as the sole energy source, respectively. When grown with thiosulfate or sodium sulfide, *Hydrogenovibrio* bacteria produced colloidal elemental sulfur, which was usually influenced by culture conditions such as the pH of the medium, which was consistent with previous reports (Javor et al., 1990; Houghton et al., 2016). Therefore, the effects of pH on the accumulation of extracellular elemental sulfur were determined in the MMJS medium, which was adjusted to pH 5.5 with citrate buffer, left neutral (pH 7.0) or adjusted to pH 8.0 with Tris-HCl buffer, respectively. The effects of oxygen concentration on the accumulation of elemental sulfur were also tested under different oxygen concentrations (2, 4, 6, 8, 10, and 20%) in the gas phase. In addition, hydrogen oxidation was tested in MMJS medium without any reduced sulfur compounds under a gas phase mixture of 80% H_2_, 18% CO_2_ and 2% O_2_ (200 kPa).

### Analysis of sulfur compounds

Thiosulfate, sulfate and sulfite were determined by ion chromatography (ICS-3000, Dionex, USA). The cultures were initially processed by centrifugation (6,000 × g, 15 min), then filtered through a 0.45 μm membrane filter as previously described (Jiang et al., 2009). Filtered samples were analyzed immediately. The sample was then purified through an analytical column (Dionex IonPac AS11-HC, 4.6 × 250 mm) using NaOH (20 mM) as an eluent. The flow rate, injection volume, column temperature and suppressor current were 0.8 ml/min, 25 μl, 30°C, and 40 mA, respectively. Elemental sulfur generated during the oxidation of thiosulfate was determined using the method developed by Li et al. (2006), which included spectrophotometric analysis for indirect quantification of sulfur produced by microorganisms. Briefly, sulfur was extracted with chloroform, then evaporated to dryness. Subsequently, the residue was dissolved in ethanol and treated with excess bisulfite to convert thiosulfate. The thiosulfate produced was then reacted with excess iodine (I_2_), after which the remaining I_2_ was determined by a spectrophotometric method.

### Electron microscopic analysis of sulfur globules

Extracellular sulfur globules were observed using scanning electron microscopy (SEM). Samples were prepared using a modified procedure from Bae et al. (2006). Cells were centrifuged at 2,000 rpm for 30 min after cultivation, then rinsed in phosphate buffer solution (PBS) (pH 7.4). The supernatant was subsequently removed and the cell pellets were fixed with 2.5% (w/v) glutaraldehyde in 0.1 M PBS (pH 7.4) for 2 h. Next, the pellets were dehydrated with ethanol stepwise with increasing concentrations (30, 50, 70, and 90%) over 10 min intervals. Finally, the cells were resuspended in absolute ethanol for 20 min. Samples were dried for 24 h to remove ethanol before SEM examination (Hitachi S-4800, Japan) at 5.0 KV.

### DNA extraction, genome sequencing, and phylogenetic analysis

Genomic DNA was extracted using the method described by Jiang et al. (2009). The quality and quantity of the extracted DNA were then determined using agarose gel electrophoresis and a NanoDrop system (Thermo NanoDrop 2000, Wilmington, Delaware, USA). The complete genome of strain S5 was sequenced by Shanghai Majorbio Biopharm Technology Co., Ltd. (Shanghai, China) using a combination of Illumina Hiseq 4000 (2 × 150 bp) and Illumina MiSeq (2 × 250 bp) platforms (Illumina, USA). Generated raw reads were first filtered to remove adapters and low-quality reads. The draft genome sequence was then assembled based on clean data generated from the Illumina Hiseq platform. The Illumina Miseq reads were used to fill in gaps, correct potential base errors and increase the consensus quality. Gaps were then filled in by sequencing the PCR products using a capillary sequencer (ABI 3730XL, ABI, USA). The complete genome sequence was assembled with SOAPdenovo (version 2.04) (http://soap.genomics.org.cn/). Gene annotation was performed by Rapid Annotation using Subsystem Technology (RAST) server (Aziz et al., 2008) and the NCBI Prokaryotic Genomes Automatic Annotation Pipeline (PGAAP). The gene functions and metabolic pathways were analyzed by searching against the Kyoto Encyclopedia of Genes and Genomes and Clusters of Orthologous Groups databases. The phylogenetic relationships of the retrieved 16S rRNA gene sequences were identified by BLAST searches (http://www.ncbi.nlm.nih.gov/BLAST) of the GenBank database. The 16S rRNA gene sequences of closely related taxa obtained from the GenBank database were aligned using CLUSTAL X1.83 (Thompson et al., 1997). Phylogenetic analysis was conducted using the MEGA 6.0 Program (Tamura et al., 2013). Distance matrices were calculated based on the Kimura two-parameter method (Kimura, 1980). Phylogenetic trees were inferred using the neighbor-joining method (Saitou and Nei, 1987) and Bootstrap values were determined based on 1,000 replications.

## Results

### Purification and morphology

Enrichment cultures were grown in liquid MMJHS medium with CO_2_ as the carbon source, thiosulfate and hydrogen as energy sources, and oxygen as a terminal electron acceptor. After 2 days of incubation, growth was observed at 28°C. The enriched culture consisted of dense populations of small, short rods and produced colloidal elemental sulfur. The cells were subsequently purified three times using the dilution-to-extinction technique at 28°C. This culture was designated as strain S5. The cells were Gram-negative with slightly curved rods about 1.5–2.5 μm long and 0.4–0.7 μm wide and motile with a polar flagellum.

### Phylogenetic analyses

**Figure 1:**
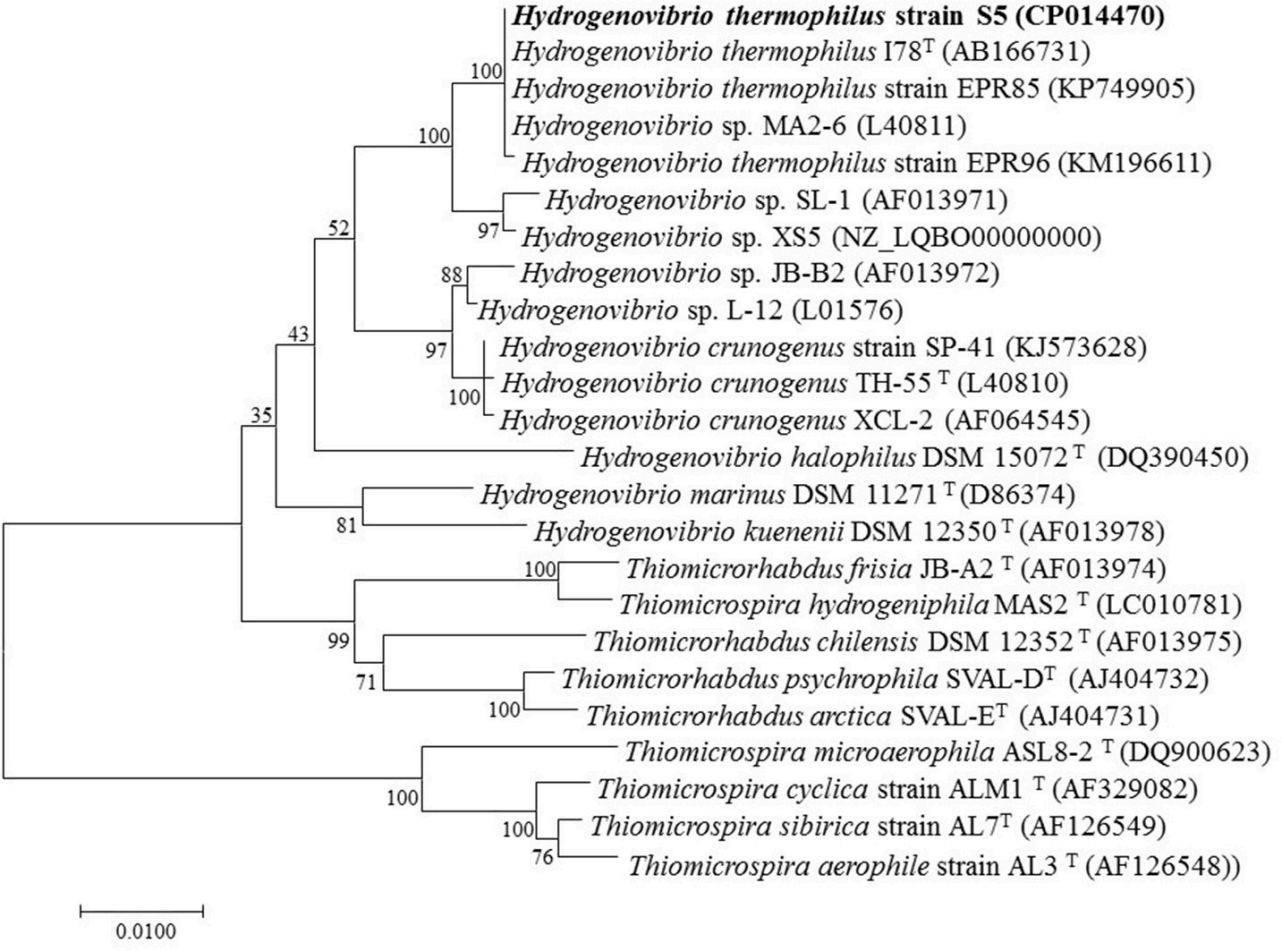
Neighbor-joining phylogenetic tree based on 16S rRNA gene sequences showing the relationships between *Hydrogenovibrio thermophilus* strain S5 and its phylogenetic neighbors. Bootstrap percentages (based on 1,000 replications) >50% are shown at the nodes. Bar = 0.01 substitutions per nucleotide position.

A BLAST search of the obtained 16S rRNA gene sequence (1,553 bp) showed that strain S5 was most closely related to *H. thermophilus* strain I78 (Takai et al., 2004; Boden et al., 2017) and *H. thermophilus* strain EPR85 (Houghton et al., 2016) with 100% 16S rRNA gene sequence similarity. Strain S5 exhibited similarities <98% with other isolates of *H. crunogenus* (Boden et al., 2017), including strain TH-55 (Jannasch et al., 1985), strain SP-41 (Hansen and Perner, 2014) and strain XCL-2 (Scott et al., 2006). The 16S rRNA gene phylogeny suggests that strain S5 clusters with bacteria of *H. thermophilus*, which was supported by a high bootstrap value of 100% (Figure 1).

### Physiological characteristics

Growth tests revealed that the isolate grew in a range of temperatures (20°C−45°C), salinities (1–4% NaCl) and oxygen concentrations (2%−20%), but that optimum growth occurred at 37°C, 4% O_2_ and 3% NaCl. No growth was observed in the absence of oxygen, and only weak growth occurred under 20% O_2_ in the gas phase, indicating that strain S5 preferred microaerobic growth conditions. After 24 h of incubation, the cells grew under both alkaline and acidic conditions, with the highest biomass occurring at pH 8.0 (2.0 × 10^8^ cells ml^−1^), followed by pH 7.0 (biomass of 1.4 × 10^8^ cells ml^−^1) and the lowest biomass (1.1 × 10^8^ cells ml^−1^) being observed for acidic cultures (pH 5.5).

The chemoautotrophic growth tests showed that strain S5 could utilize thiosulfate, sulfide, elemental sulfur, tetrathionate or thiocyanate as the sole energy source, but that it could not utilize sulfite, and elemental sulfur and thiocyanate only supported weak growth. Molecular hydrogen could also be used as an energy source. Heterotrophic growth tests showed that strain S5 was unable to grow using any of the tested organic carbons as the sole energy source, but when reduced sulfur compounds such as thiosulfate were present it grew with various organic carbons sources, including each kind of 20 amino acids, tryptone, yeast extract, casamino acids, casein, acetate, formate, citrate, propionate, tartrate, succinate, glucose, and fructose. Relatively high biomasses were obtained when cells utilized tryptone, methionine, arginine, glutamate or hydroxyproline as a carbon source, but the biomasses were less than those obtained under chemoautotrophic conditions. Other organic compounds, including peptone, starch, pyruvate, sucrose, galactose, lactose maltose and trehalose, failed to support its growth. These results confirm that strain S5 prefers a chemolithoautotrophic lifestyle.

### Oxidation of reduced sulfur compounds

**Figure 2:**
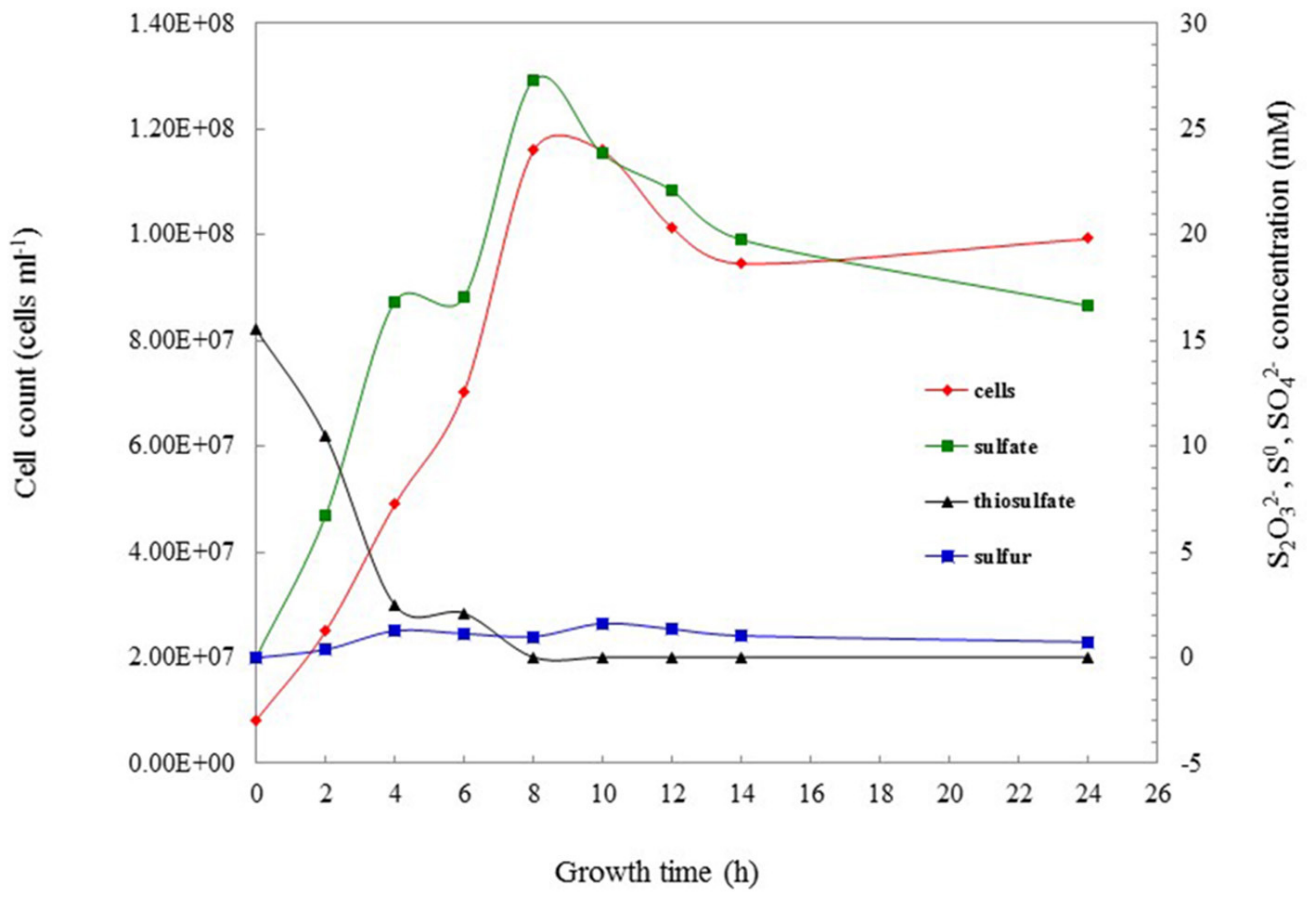
The thiosulfate-oxidizing characterization of *Hydrogenovibrio thermophilus* strain S5 under optimal culture conditions. Data show the oxidation of thiosulfate (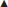), the production of sulfate (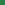) and sulfur (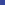), and concomitant bacterial growth (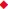) of strain S5.

The growth on reduced sulfur compounds was further determined in more detail using different concentrations of thiosulfate (5, 10, 15, 20, 30 mM) and sulfide (50, 100, 200, 400, 800 μM, 1, 2 mM) as sole energy sources. Strain S5 grew with thiosulfate at all tested concentrations from 5 to 30 mM, with 10 mM supporting maximal growth (1.16 × 10^8^ cells ml^−1^). Additionally, growth was observed in the presence of sulfide at 1 mM and below, with optimal growth (1.19 × 10^8^ cells ml^−1^) occurring in the presence of 100 μM sodium sulfide. No growth was observed when the sulfide concentration was 2 mM. The growth rate and sulfur oxidation rate were 0.35 h^−1^ and 1.045 mM h^−1^, respectively, under optimal cultivation conditions with 10 mM thiosulfate (Figure 2). When strain S5 grew at pH 5.5 (in buffered medium) and pH 7.0 (unbuffered), elemental sulfur accumulated in the culture. These findings suggested that strain S5 performed incomplete thiosulfate oxidation, while sulfite was not detected as an intermediate during this process. However, alkaline conditions lead to further oxidation of elemental sulfur, such as at pH 8.0. Elemental sulfur generation from thiosulfate was also affected by oxygen concentrations in the gas phase. The maximum accumulation of elemental sulfur occurred in 4% oxygen, which was also the optimal condition for growth.

**Figure 3:**
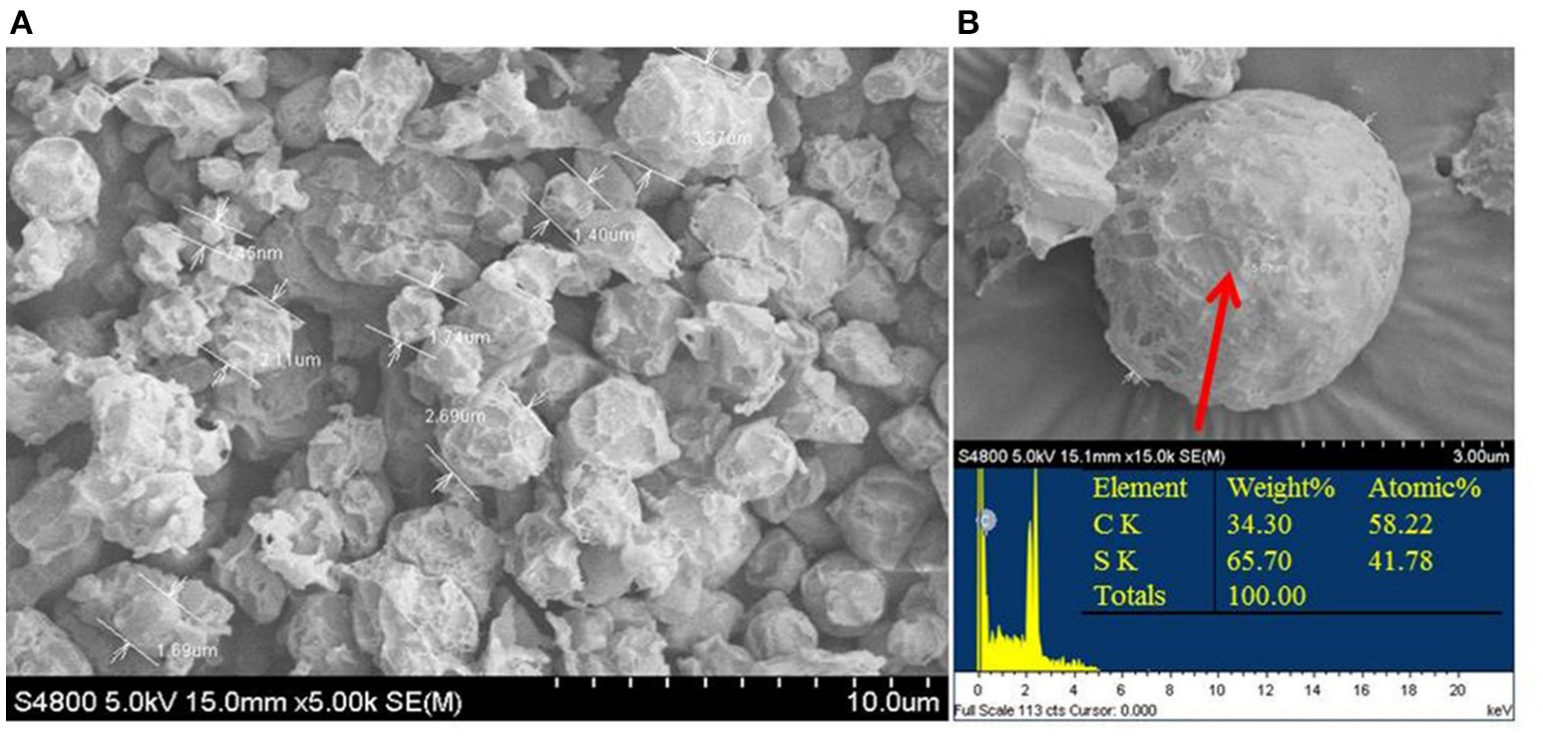
Scanning electron micrograph **(A)** and energy-spectrum analysis **(B)** of extracellular sulfur globules produced by *Hydrogenovibrio thermophilus* strain S5 during incomplete oxidation of thiosulfate.

Elemental sulfur occurred in the culture at the start of the mid-exponential growth phase, but more accumulated at the end of the exponential phase and during the stationary phase (Figure 2). Morphological observations under SEM showed that elemental sulfur was in the form of globules outside the cell, with the size varying from 700 nm to 6.0 μm (Figure 3). Energy dispersive X-ray spectroscopic analysis further revealed that the sulfur globules mainly contained carbon and sulfur. Elemental sulfur was the main component, accounting for 51.5–71.3% of the total (Figure 3).

### Genomic features

The complete genome of strain S5 comprised 2.77 Mb with a G+C content of 50.52 mol%, which was remarkably higher than that of three other *Hydrogenovibrio* bacteria (Table **2**). Gene annotation based on RAST predicted 2598 genes, including 2463 protein-encoding genes, 49 tRNA-encoding genes and 12 rRNA-encoding genes. We compared the genomes of closely related species available in public databases, including strains XCL-2 (Scott et al., 2006), MA2-6 (unpublished data) and XS5 (Zhang et al., 2016). The genome of strain S5 was similar to those of strains MA2-6 and XS5 in G+C content and coding density, but distinctly different from that of strain XCL-2 (Table **2**). The average nucleotide identity (ANI) between strain S5 and other *Hydrogenovibrio* bacteria was similar at the genome level (Table **2**). The ANI of strain S5 with strain MA2-6 was highest (96.94%), followed by that of strain XS5 (90.10%), and a much lower ANI (73.17%) with strain XCL-2 (Table **2**), indicating that the genome of strain S5 was most similar to that of strain MA2-6.

### Central metabolism

#### Oxidation of sulfur and hydrogen

The genome of strain S5 contained homologs for genes *soxAYXBCD*, all of which are necessary for assembling a Sox system that oxidizes reduced sulfur compounds (Table **2**). Strangely, the *soxZ* gene is absent in the strain S5 genome, but present in all other members of this genus (Table **2**). Genes encoding the Sox components are not organized in a single cluster in strain S5, which seems common in this genus (Table **2**). Strain S5 also contained a homolog of the sqr gene, which usually oxidizes sulfide to elemental sulfur, resulting in the deposition of sulfur outside the cells. Unlike *H. crunogens* strain XCL-2, strain S5 contained more genes involved in sulfur metabolism, including flavocytochrome-C sulfide dehydrogenase (FCC), intracellular sulfur oxidation protein (*DsrEF*), sulfite reductase (*CysIJ*) and sulfate adenylyltransferase (*CysDH*) (Table **2**). All genes essential for the assimilation of sulfate reduction were present in the genome of strain S5. No genes involved in dissimilatory sulfate reduction, such as sulfite reductase (*DsrAB*), electron transfer protein (*DsrC*) or membrane-bound electron-transporting complex (*DsrMKJOP*), were detected in this bacterium (Table **2**).

Genomic analysis revealed that strain S5 contained two hydrogenase genes, [NiFe]-Hydrogenases Group 1 (*hyaAB*) and Group 2b (*hupUV*), which were organized into two operons. According to a recent classification of the hydrogenases in the genus *Thiomicrospira* (Hansen and Perner, 2016), Group 1 hydrogenase of strain S5 can be further categorized into cluster I, together with those from strain MA2-6 and *H. marinmus*. These were distinctly different from cluster II hydrogenases, which exclusively contains those from *H. crunogenus* (Table **2**). In addition, Group 2b hydrogenase was also detected in strain MA2-6, but absent from the genome of *H. crunogens* XCL-2. Neither [NiFe]-Hydrogenases Group 1 nor Group 2b was found in the non-vent bacterium strain XS5.

#### Carbon metabolism

All enzymes essential for central carbon metabolism are encoded by strain S5 (Table **2**). Strain S5 was most similar to strain XS5 with respect to CO_2_ fixation, and not its closest bacterium strain MA2-6 (Table **2**). Like other members of the genus *Hydrogenovibrio*, strain S5 has a carbon-concentrating mechanism that facilitates the uptake of dissolved inorganic carbon. Similar to strains MA2-6 and XS-5, strain S5 possesses three types of carbonic anhydrases (α-, β-, and γ-class). This contrasts with *H. crunogenus* strain XCL-2, which only has two classes (α- and β-class) (Table **2**). Moreover, strain S5 contains all genes encoding enzymes involved in the CBB cycle. Glycolysis and gluconeogenesis, the tricarboxylic acid cycle and the pentose phosphate pathway are also complete in this genome. Two pathways for acetate assimilation are present in the genome of strain S5: (1) a two-step reaction including acetate kinase and phosphate acetyltransferase and (2) a single step reaction including acetyl coenzyme A synthetase. Strain S5 also contains an acetate permease (*ActP*) function as an acetate transporter. In contrast, only acetyl coenzyme A synthetase for acetate assimilation was found in the genome of *H. crunogenus* XCL-2.

#### Respiration

For aerobic respiration, strain S5 contains genes encoding complexes I–IV of the respiratory chain, including NADH ubiquinone oxidoreductase (*NuoABCDEFGHIJKLMN*; complex I), succinate dehydrogenase (*FrdABC*; complex II), cytochrome *bc1* complex (*PetABC*; complex III), cytochrome *c* oxidoreductase (*CcoNOP*; complex IV). In addition, strain S5 has a bd-type cytochrome oxidase (*CydAB*), which is absent in *H. crunogenus* XCL-2 (Table **2**). A low oxygen adapted cbb3-type cytochrome *c* oxidase was also detected in strain S5. The presence of genes *atpABCDEFGH* in this genome indicates that the respiratory chain is linked to a F0F1 ATPase that generates ATP. Strain S5 cannot utilize nitrate as an electron acceptor based on physiological analyses. Similarly, no genes encoding nitrate reductase (*NasA*) or nitrite reductase (*NirBD*) were found in this genome or in strain MA2-6. However, both the *nasA* and *nirBD* genes were present in the genomes of the other two isolates (Table **2**).

## Discussion

*Hydrogenovibrio* bacteria, previously described as members of the genus *Thiomicrospira*, have frequently been found surrounding deep-sea hydrothermal vents (Ruby and Jannasch, 1982; Jannasch et al., 1985; Brinkhoff and Muyzer, 1997; Wirsen et al., 1998; Ahmad et al., 1999; Takai et al., 2004; Brazelton and Baross, 2010; Cao et al., 2014; Hansen and Perner, 2014; Houghton et al., 2016). Our previous study, which was based on metagenomics, revealed that this group was one of the dominant populations in hydrothermal vent chimneys of the SWIR (Cao et al., 2014). These organisms likely play an important role in the biogeochemical cycles of sulfur; however, the mechanisms underlying their adaptations to such harsh environments remain unclear. In this study, a new strain was isolated from a sulfide chimney in the SWIR. On the basis of 16S rRNA gene phylogeny, physiological and genomic properties, the newly isolated strain was designated as *Hydrogenovibrio thermophilus* strain S5, and further subjected to characterization of sulfur oxidation and environmental adaptations. The results of this study highlight the specifications of *Hydrogenovibrio* bacteria to adapt to their vent niches, and imply their roles in the carbon and sulfur cycles of deep-sea hydrothermal environments.

### Adaptation of *Hydrogenovibrio* bacteria to deep-sea hydrothermal vents

With the exception of *H. thermophilus* strain I78, which grows at 55°C, most *Hydrogenovibrio* isolates cannot tolerate high temperatures and usually grow at below 40°C (Table 1). It has been implied that *Hydrogenovibrio* bacteria preferentially inhabit the mesophilic mixing zones of hydrothermal vents. Moreover, thermodynamic calculations have shown that the oxidation of sulfide is most favorable at relatively low temperatures (McCollom and Shock, 1997). Another key trait of deep-sea *Hydrogenovibrio* bacteria that facilitates its adaptation to vent environments is the requirement of oxygen for growth. Indeed, these organisms were all described as microaerobic bacteria and incapable of growth under anaerobic conditions (Table 1). Genomic analyses confirmed that sulfur-oxidizing pathways in all of the available genome-sequenced bacteria of this genus require O_2_ as a terminal electron acceptor. Moreover, strain S5 possesses two cytochrome oxidases, a cbb_3_-type and a bd-type, which have been proposed to have a high affinity for O_2_ and therefore play an important role in microaerobic growth (D'Mello et al., 1996; Preisig et al., 1996). Thus, the metabolically habitable areas of *Hydrogenovibrio* bacteria strictly require O_2_. *H. thermophilus* strain S5 can adapt to a wide range of oxygen concentrations, ranging from 2 to 20%, with an optimum of 4% (Table 1). The optimal growth pH of strain S5 was pH 8.0, which is consistent with seawater pH, and similar to that of other deep-sea *Hydrogenovibrio* isolates (Wirsen et al., 1998; Takai et al., 2004). However, the optimal growth of strain I78 occurred at pH 6.0 (Table 1), even though strain S5 showed the highest 16S rRNA gene sequence similarity (100%) to strain I78 (Takai et al., 2004). Moreover, pH significantly affected the sulfur-oxidizing process of strain S5. At higher pH, strain S5 completely oxidized thiosulfate to sulfate, while thiosulfate was oxidized to elemental sulfur deposits outside of the cells under acidic conditions. The phenomenon was also observed in *H. crunogenus* (Javor et al., 1990) and *H. thermophilus* (Houghton et al., 2016). The vent fluids are typically acidic in basalt-hosted hydrothermal vents; hence, it is likely that *Hydrogenovibrio* species can generate elemental sulfur globules *in situ*.

**Table 1:** Characteristics differentiating *Hydrogenovibrio thermophilus* strain S5 from other strains within the genus *Hydrogenovibrio* from different deep-sea hydrothermal vents.

**Characteristics**	**1**	**2**	**3**	**4**	**5**
**Source of the isolates**	**Active hydrothermal chimney, Southwest Indian Ridge**	**Hydrothermal diffusing flow, Western Pacific**	**Vestimentiferan tube worm, East Pacific**	**Mussel periostracum, Galapagos Rift, East Pacific**	**Polymetal sulfide rock, Mid-Atlantic Ridge**
Morphology	Slightly curved rods; 0.4~0.7 ^*^1.5~2.5 μm	Straight to curved rods; 0.4~0.7 ^*^0.8~1.5 μm	Vibrioid; 0.4~0.5 ^*^1.5~3.0μm	Comma to spiral shape; 0.3 ^*^2.0~3.0 μm	Slightly vibrioid; 0.5~0.7 ^*^1.3~2.0 μm
Motility	+	+	+	_+_	+
Optimal temperature (°C)	37	35–40	28–32	25	28–32
Optimal pH	8.0	6.0	7.5–8.0	8.0	7.5
O_2_ concentration for growth (%):Optimum (upper limit)	4 (20)	0.5–1.0 (10)	Microaerobic (>20)	Microaerobic (>20)	Microaerobic (>20)
Maximum growth rate (h^−1^)	0.35	0.69	0.8	0.32	0.8
**ENERGY SOURCE**					
Thiosulfate	+	+	+	+	+
Sulfide	+	+	+	+	+
Elemental sulfur	+	+	+	+	+
Sulfite	–	–	–	–	–
Thiocyanate	+	ND	–	–	ND
Tetrathionate	+	+	ND	+	ND
Hydrogen	+	–	+	+	+
Accumulation of S^0^	+	+	+	+	+
Utilization of organic compound as carbon sources	+	+	+	+	–
G+C content (mol %)	51.50%	43.8%	44.2%	44.4± 0.2%	44.6 ± 0.3%

*Strains: 1, strain S5; 2, Hydrogenovibrio thermophilus strain I78 (Takai et al., 2004); 3, Hydrogenovibrio crunogenus strain TH-55 (Jannasch et al., 1985); 4, Thiomicrospira sp. strain L-12 (Ruby and Jannasch, 1982); 5, Thiomicrospira sp. strain MA-3 (Wirsen et al., 1998). +, positive; –, negative*.

*H. thermophilus* strain S5 can utilize a wide range of inorganic sulfur compounds, including sulfide and partially oxidized inorganic sulfur compounds (e.g., thiosulfate, elemental sulfur, and tetrathionate) (Table 1) and prefer moderately low concentrations of sulfide (0.1–1 mM). However, it cannot grow under hydrogen sulfide concentrations of 2 mM or greater, although it can oxidize sulfide to elemental sulfur for detoxification. Hydrogen sulfide is usually rich in the vent fluids of black hydrothermal chimneys, with concentrations typically in the millimolar range (Martin et al., 2008), while partially oxidized inorganic sulfur compounds, such as elemental sulfur, thiosulfate and polysulfide, occur in the areas around the vent and outer layers of the chimneys where the fluid mixes with oxygenic water. The utilization of partially oxidized inorganic sulfur compounds provides ecological advantages for strain S5, which could mitigate exposure to high temperatures and toxic chemicals in venting fluids.

In addition to inorganic sulfur compounds, *Hydrogenovibrio* species can also utilize H_2_ and Fe^2+^ as energy sources to fuel carbon fixation (Hansen and Perner, 2016; Barco et al., 2017). This versatile energy metabolism could enable these organisms to be widespread and highly adaptable at inhabiting different hydrothermal vent fields globally, including the East Pacific Rise, Pacific Ocean and SWIR. Comparison of strain S5 with other *Hydrogenovibrio* isolates revealed its physiological features (Table 1) and genomic characteristics (Table 2) in energy sources, optimal O_2_ concentration for growth, sulfur-oxidizing genes, and hydrogenase. The results further demonstrated the specification and adaptation of *Hydrogenovibrio* bacteria to different hydrothermal vent environments.

**Table 2:** Comparison of the genomes between *Hydrogenovibrio thermophilus* strain S5 and its closest relatives based on RAST annotations in this study.

	***Hydrogenovibrio thermophilus* strain S5**	***Hydrogenovibrio crunogenus* strain XCL-2**	***Hydrogenovibrio* sp. strain MA2-6**	***Hydrogenovibrio* sp. strain XS5**
**Isolated source**	**Hydrothermal vent environment in Southwest Indian Ridge**	**Hydrothermal vent environment in Pacific Ocean**	**Hydrothermal vent environment in Mid-Atlantic Ridge**	**Brine-seawater Interface of Kebrit Deep in the Red Sea**
Genome size (Mb)	2.77	2.43	2.68	2.63
G+C content (%)	50.5	43.1	50.1	50.1
Coding density (%)	89.0	90.3	88.3	89.3
ANI		73.17	96.94	90.10
**SULFUR OXIDATION**				
Sox	SoxCD; SoxAYX; soxB; SoxB	SoxCD; SoxAZYX; SoxB	SoxCD; SoxAZYX; SoxB; SoxXA, SoxB	SoxCD; SoxAZYX; SoxB; SoxB
Sulfide:quinone oxidoreductase (SQR)	+	+	+	+
Flavocytochrome-c sulfide dehydrogenase (FCC)	+	–	+	+
Intracellular sulfur oxidation protein (DsrEF)	+	–	+	+
**ASSIMILATORY SULFATE REDUCTION**				
Sulfite reductase (CysIJ)	+	–	+	–
Sulfate adenylyltransferase (CysDH)	+	–	+	–
**HYDROGENASE**				
[NiFe]-Hydrogenases Group 1	Cluster I	Cluster II	Cluster I	–
[NiFe]-Hydrogenases Group 2b	+	–	+	–
**CARBON METABOLISM**				
Carbonic anhydrase	1α-class, 1β-class, 2 γ-class	1α-class, 2 β-class	2 α-class, 2 β-class, 1 γ-class	1α-class, 1β-class, 2 γ-class
Calvin-Benson cycle	+	+	+	+
Glycolysis/Gluconeogenesis	+	+	+	+
Pentose phosphate pathway	+	+	+	+
Acetate kinase	+	–	+	+
Phosphate acetyltransferase	+	–	+	+
TCA	+	+	+	+
GDP-mannose mannosyl hydrolase	–	–	+	–
**CYANATE DEGRADATION**				
Cyanate ABC transporter	+	–	+	–
Cyanate hydratase (CynS)	+	+	+	+
**NITRATE AND NITRITE AMMONIFICATION**				
Nitrate reductase (NasA)	–	+	–	+
Nitrite reductase (NirBD)	–	+	–	+
**OXYGEN REDUCTION**				
NADH ubiquinone oxidoreductase (NuoABCDEFGHIJKLMN)	+	+	+	+
Succinate dehydrogenase (FrdABC)	+	+	+	+
Cytochrome *bc1* complex (PetABC)	+	+	+	+
Cytochrome c oxidoreductase (CcoNOP)	+	+	+	+
Cytochrome d ubiquinol oxidases (CydAB)	+	–	+	+

*The complete genome sequence of Hydrogenovibrio crunogenus strain XCL-2 (GenBank accession number: NC_007520), and the draft genome sequences of Hydrogenovibrio sp. strain MA2-6 (GenBank accession number: NZ_JOMK01000001) and Hydrogenovibrio sp. strain XS5 (GenBank accession number: LQBO00000000) are available in the NCBI database. +, present; –, absent*.

### Sulfur oxidation pathways in *H. thermophilus* S5

**Figure 4:**
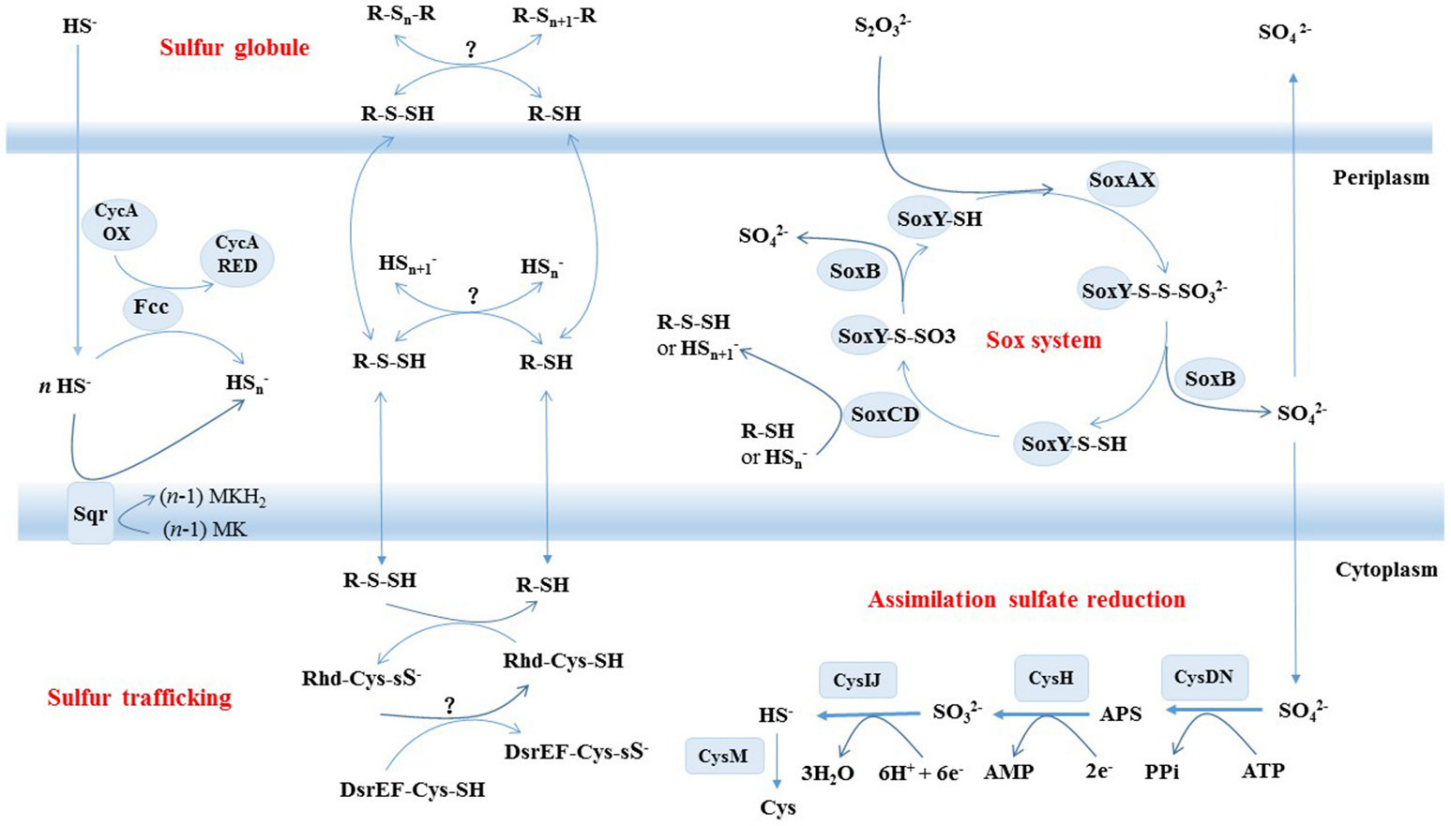
Schematic illustration of putative pathways of sulfur metabolism in *Hydrogenovibrio thermophilus* strain S5. All oxidative enzyme systems shown in the periplasm (SOX, SQR, and FCC) and extracellular sulfur globules contribute to a putative pool of oligosulfides (HS_n_ and possibly organic R-S_n_-H). Putative sulfur transfer reactions and assimilatory sulfate reduction are proposed to occur in cytoplasm.

To examine the metabolic pathways of sulfur oxidation, we generated the complete genome sequence of strain S5. Prior to this study, the only member from deep-sea hydrothermal vents to have its entire genome sequenced was *H. crunogenus* strain XCL-2 (Scott et al., 2006), which is distantly related phylogenetically and also significantly differs in physiological traits from strain S5 (Tables 1, 2). Based on the genome data and growth tests, we drew a schematic model of sulfur oxidation in *Hydrogenovibrio thermophilus* strain S5 (Figure 4). Hydrogen sulfide is oxidized to elemental sulfur by two pathways, namely SQR and FCC. SQR transfers electrons from sulfide to the respiratory chain via quinone, while FCC donates electrons via cytochrome *c* (Chen et al., 1994; Visser et al., 1997; Griesbeck et al., 2000). SQR plays a more important role than FCC in phototrophic and chemotrophic sulfide oxidation (Chan et al., 2009). Moreover, SQR was found to be involved in sulfide detoxification. Therefore, it was presumed that SQR could play a more important role than FCC during sulfide oxidation in hydrothermal vents. However, FCC has a high affinity for sulfide, which might be an advantage for cells under very low concentrations of sulfide (Chan et al., 2009). Thiosulfate can be oxidized by a Sox system (*SoxABCDXY*) in the periplasm of strain S5. Despite the absence of the *soxZ* gene, strain S5 is still capable of completely oxidizing reduced sulfur compounds to sulfate. *SoxZ* is usually combined with *SoxY* as a heterodimeric carried protein (*SoxYZ*) to shuttle covalently attached intermediates between the enzymes of the Sox pathway (Grabarczyk et al., 2015), which is present in all other genomes of this genus (Table 2), which implies that *SoxZ* is not essential for strain S5 to oxidize thiosulfate. Incomplete oxidation of thiosulfate will lead to the formation of extracellular elemental sulfur, which was also observed in *H. thermophilus* (Houghton et al., 2016). Usually, *SoxCD*-containing bacteria cannot generate extracellular sulfur during thiosulfate oxidation; however, *Hydrogenovibrio* bacteria from deep-sea hydrothermal vent systems seem to be an exception. Elemental sulfur produced by the incomplete oxidation of sulfide or thiosulfate under lower pH was excreted outside of the cells of *H. thermophilus* strain S5 and formed sulfur globules (Figure 3). Generally, elemental sulfur produced in the periplasm can also be transported into the cytoplasm for further oxidation, which is usually performed by the dissimilatory sulfite reductase (*DsrAB*) or a heterodisulfide reductase (*Hdr*)-like enzyme (Dahl, 2009; Gregersen et al., 2011). Moreover, sulfur carrier proteins *Rhd, TusA* and *DsrEFH* were found to be involved in sulfur transfer from persulfide intermediates to *DsrAB* (Stockdreher et al., 2014). The protein *DsrEFH* occurs exclusively in sulfur oxidizers and is essential for the oxidation of sulfur stored in the intracellular sulfur globules of purple sulfur bacteria, such as *Allochromatium vinosum* (Dahl et al., 2008). In this study, the *Dsr* system and *hdr* genes were not detected in this genome. However, the homologs of *rhd* and *dsrEF* genes were first discovered in *Hydrogenovibrio* isolates (Table 2). Taken together, these findings indicate that an unknown sulfur trafficking process might be present in this genus.

Extracellular sulfur globules are generated by a diverse group of chemotrophic and phototrophic bacteria (Dahl and Prange, 2006). However, few studies have investigated extracellular sulfur globules produced by chemoautotrophs (Prange et al., 2002; Dahl and Prange, 2006; Beard et al., 2011). In this study, the properties of sulfur globules secreted by *H. thermophilus* strain S5 cells were analyzed by SEM and EDX. This is the first report detailing sulfur globules from *Hydrogenovibrio* bacteria, and also the first report evaluating chemoautotrophic sulfur-oxidizing bacteria from deep-sea hydrothermal vents.

### Assimilatory sulfate reduction in *H. thermophilus* S5

All known genes involved in assimilatory sulfate reduction were present in *H. thermophilus* strain S5. Sulfate uptake is performed by two sulfate permeases: (1) one belonging to the ArsB/NhaD superfamily and (2) another belonging to a high-affinity transporter *SulP*. The *ArsB/NhaD* permease is located in the immediate vicinity of gene clusters involved in assimilatory sulfate reduction. Therefore, the transporter might be involved in sulfate transport for sulfate assimilation. Once inside the cell, the sulfate is activated to form adenylyl sulfate (APS), which is catalyzed by the enzyme ATP sulfurylase (*CysDN*). Subsequently, strain S5 directly reduces APS to sulfite by APS reductase (*CysH*) without further phosphorylation to phosphoadenosine-5'-phosphosulfate (PAPS) as an intermediate. The generated sulfite is further reduced to sulfide by an assimilatory sulfite reductase (*CysIJ*), which is quite distantly related to their dissimilatory counterparts (*DsrAB*). The final step is catalyzed by O-acetylserine (thiol)-lyase B (*CysM*), which synthesizes cysteine from O-acetylserine and sulfide. In addition, some studies have indicated that *CysM* could also utilize thiosulfate to produce cysteine (Kredich, 1992; Sievert et al., 2008). Hence, it is possible that the *CysM* in strain S5 performs a similar function for thiosulfate assimilation. This is the first report of the assimilatory sulfate reduction pathway in the genus *Hydrogenovibrio*. Assimilatory sulfate reduction enables microorganisms to reduce sulfate for the formation of amino acids, nucleic acids and sulfur-containing coenzymes. The result suggests that strain S5 is more versatile in sulfur sources than other members of this genus that do not have an assimilatory sulfate reduction pathway. Specifically, strain S5 can reduce sulfate and/or thiosulfate for the synthesis of cellular material when reduced sulfur compounds are limited in deep-sea hydrothermal vents.

## Conclusions

*H. thermophilus* strain S5 has specifically adapted itself to hydrothermal vent environments, mainly via sulfur oxidation and carbon dioxide fixation. This organism seems to prefer deep-sea vent niches with moderate temperatures (37°C), alkaline pH (pH 8.0), microaerobic conditions (4% O_2_), various reduced sulfur compounds (e.g., 100 μM of sulfide) and H_2_. Furthermore, it adapts well to vent environments with steep physical and chemical gradients, where varied inorganic sulfur compounds, low concentrations of carbon dioxide, changing oxygen concentrations and pH values usually occur. A schematic model of sulfur metabolism in strain S5 was generated based on genomic analysis, providing the first detailed description of the genus *Hydrogenovibrio*. An assimilatory sulfate reduction pathway was found in the genus *Hydrogenovibrio* for the first time, which might support their growth far from the vent, where sulfide is depleted. In addition, the properties of extracellular sulfur globules, including their morphology, size and elemental composition, were documented in *Hydrogenovibrio* bacteria. The activity of *H. thermophilus* strain S5 in sulfur oxidation and the formation of extracellular sulfur globules highlight its role in sulfur cycling in deep-sea hydrothermal vent environments.

## Author contributions

LJ designed the study, analyzed the data and wrote the manuscript. JL did experiments, analyzed the data and wrote the manuscript. ZS designed the study and wrote the manuscript.

### Conflict of interest statement

The authors declare that the research was conducted in the absence of any commercial or financial relationships that could be construed as a potential conflict of interest.
